# Associations of Physical Activity and Handgrip Strength with Different Domains of Quality of Life in Pediatric Cancer Survivors

**DOI:** 10.3390/cancers14102554

**Published:** 2022-05-23

**Authors:** Ankie Tan Cheung, William Ho Cheung Li, Laurie Long Kwan Ho, Wei Xia, Yuanhui Luo, Godfrey Chi Fung Chan, Joyce Oi Kwan Chung

**Affiliations:** 1 The Nethersole School of Nursing, Faculty of Medicine, The Chinese University of Hong Kong, New Territories, Hong Kong, China; ankiecheung@cuhk.edu.hk (A.T.C.); longkwanho@cuhk.edu.hk (L.L.K.H.); 2 School of Nursing, Sun-Yat-sen University, Guangzhou 510080, China; xiaw23@mail.sysu.edu; 3 Xiangya School of Nursing, Central South University, Changsha 410013, China; yuanhui3@csu.edu.cn; 4 Department of Pediatric Oncology, Hong Kong Children’s Hospital, Kowloon, Hong Kong, China; gcfchan@hku.hk; 5 School of Nursing, The Hong Kong Polytechnic University, Kowloon, Hong Kong, China; okjoyce.chung@polyu.edu.hk

**Keywords:** cancer, children, handgrip strength, pediatric, physical activity, quality of life, survivors

## Abstract

**Simple Summary:**

Physical activity has been targeted as a leading priority for health promotion in pediatric survivorship care. It is unclear whether regular physical activity can enhance the quality of life among Chinese pediatric cancer survivors. We researched this question in our study of 191 survivors of pediatric cancer. We found that the survivors who engaged in higher levels of physical activity reported higher scores across all domains of quality of life (i.e., physical, emotional, social, and school functioning). We also observed that the survivors with higher PA levels exhibited greater handgrip strength than the survivors with lower PA levels. Notably, the survivors with greater handgrip strength also had better physical and emotional functioning.

**Abstract:**

There is a paucity of evidence about the associations of physical activity (PA) and handgrip strength (HGS) within different domains of quality of life (QoL) in Chinese pediatric cancer survivors. We, therefore, conducted this multicenter cross-sectional study aimed to investigate whether increased PA level and HGS are associated with higher scores in different QoL domains (i.e., physical, emotional, social, and school functioning) in pediatric cancer survivors. PA was assessed with a validated self-reported PA rating scale. In total, 191 Chinese pediatric cancer survivors aged 9 to 16 years were included in the analysis. Results showed that engaging in a higher level of PA was significantly associated with improved QoL in different domains, including physical (*β* = 0.543, *p* < 0.001), emotional (*β* = 0.449, *p* < 0.001), social (*β* = 0.434, *p* < 0.001), and school functioning (*β* = 0.407, *p* < 0.001). Greater HGS was also associated with better physical (*β* = 0.230, *p* ≤ 0.001) and emotional (*β* = 0.261, *p* ≤ 0.001) functioning. Findings from this study provide evidence of the significant beneficial impact of regular PA on pediatric cancer survivors’ QoL along their survivorship trajectory.

## 1. Introduction

Pediatric cancer is a leading cause of death in children and adolescents [1]. Every year, approximately 400,000 children and adolescents under the age of 20 years are diagnosed with cancer worldwide [2]. Provided the continuous advances in early detection and treatment of pediatric cancer, the overall 5-year survival rate is currently around 85% [2,3]. Such an encouraging survival rate indicates that there is a growing population of pediatric cancer survivors living with significant medical and psychological morbidity associated with curative therapies [4,5,6]. Studies have reported that between 60% and 90% of pediatric cancer survivors experience at least one chronic health condition in their survivorship trajectory [7,8]. The risk of developing late effects increases with the elapsed time from cancer diagnosis [7,8]. The presence of serious and disabling late effects poses a significant threat to the health status of survivors, leading to functional impairment and activity limitations [9,10], hence jeopardizing their quality of life (QoL) [4].

QoL has been found to be a strong predictor of survival among cancer patients [11]. It is thus a clinically important outcome emphasized in survivorship oncology research [12]. The World Health Organization (WHO) defines QoL as the perception of one’s position in life embedded in a cultural, social, and environmental context [13]. It is a multidimensional construct that encompasses physical, mental, emotional, and social functioning domains. Evaluation of these domains can reveal the overall impact of their disease and treatment on survivors’ general functioning. Evidence shows that pediatric cancer survivors reported significantly lower QoL than their healthy siblings and counterparts [14,15]. Attention has been increasingly placed on exploring effective approaches to improving QoL in pediatric cancer survivors, particularly those health-enhancing behaviors.

Physical activity (PA), a modifiable health-enhancing behavior, has emerged as a priority concern in pediatric oncology survivorship research [16]. Accumulating evidence shows that PA may attenuate many cancer-related late effects experienced by pediatric cancer patients and survivors, with improvement indicated in a wide array of health outcomes, thereby improving QoL [17,18,19,20]. However, most evidence is based on the Western survivors, whose prognosis might differ from survivors in Asia due to differences in body composition and metabolic profile [21]. In addition, all of the aforementioned studies focus on adolescents [17], young adults, [18] or adult cancer survivors [19], all of whom have significant differences in growth and developmental stages compared with children who have survived cancer. Some studies target pediatric cancer patients who are undergoing cancer treatment [18,20], whose experiences and perceptions might differ significantly from those of pediatric cancer survivors [22]. Hence, findings from these studies cannot be generalized to Chinese children and adolescents who have survived cancer. Therefore, the role of PA in different key domains of QoL in Chinese pediatric cancer survivors remains unclear.

Handgrip strength (HGS), a simple and reliable measure of evaluating physical functioning, has been supported as a biomarker of overall health status and QoL in aging and diverse clinical populations [23,24,25,26,27]. Some evidence shows that HGS can predict QoL in various groups of people with diseases, ranging from people with chronic liver diseases [24] to adults with depression [26] and children and adolescents with idiopathic arthritis [27]. Yet, data on the impact of HGS on QoL among pediatric cancer survivors are lacking.

Recognizing the importance of improving QoL and health status in this vulnerable group, there is a pressing need to expand our understanding of the above-mentioned under-researched areas to develop effective clinical interventions for Chinese pediatric cancer survivors. The aim of the present study was to investigate the association of QoL with PA and HGS among Chinese pediatric cancer survivors. Specifically, we hypothesized that higher participation in PA and greater HGS are associated with better QoL in different domains (i.e., physical, emotional, social, and school functioning) among survivors of pediatric cancer.

## 2. Materials and Methods

### 2.1. Study Design and Participants

We conducted a multicenter cross-sectional study on pediatric cancer survivors attending pediatric oncology outpatient clinics in two public hospitals in Hong Kong. Pediatric cancer survivors who met the following inclusion criteria were invited to participate in the study: (1) Chinese pediatric cancer survivors between 9 and 16 years old (on assumption that survivors younger than 9 may be perplexed by questionnaires due to limited cognitive processing and task performance skills); (2) survivors who had completed treatment at least 6 months ago; and (3) survivors who were able to communicate in Cantonese/Mandarin and read Chinese. The exclusion criteria were: (1) survivors with evidence of recurrence or second malignancies; (2) survivors who could not fill out questionnaires independently due to cognitive impairment; and (3) survivors with significant physical or mental impairment identified in their clinical records.

### 2.2. Sample Size Calculation

With reference to previous studies [17,18,19,20,21], we aimed to detect a small to medium effect size (R^2^ = 0.04; Cohen’s *f*^2^ = 0.042 [28]) of the association between physical activity and health-related quality of life. By using the power analysis software (G * Power 3.1 [29]), it was estimated that a sample size of 191 participants would be adequate to detect an R^2^ change of at least 0.04 with 80% power at 5% level of significance using multiple regression (i.e., if physical activity level explains at least 4% of variance of health-related quality of life after adjusting for demographic and clinical characteristics, it would be detectable with 80% power at 5% level of significance)

### 2.3. Data Collection Procedures

Eligible pediatric cancer survivors were recruited between March 2019 and January 2021 from two pediatric oncology outpatient clinics in Hong Kong. Prior to the commencement of the study, ethical approval was obtained from the institutional review board of the University of Hong Kong/Hospital Authority Hong Kong West Cluster (UW 19-053) and Hong Kong Children’s Hospital (HKCH-REC-2019-017). At enrollment, an information sheet explaining the purpose and nature of the study and associated potential benefits and harms was provided for the children and their parents/guardians. Thereafter, parents/guardians provided written informed consent for their children, and the children assented by writing their names on a specially designed assent form and completing the standardized questionnaires. The study adhered strictly to the principles of the Declaration of Helsinki.

### 2.4. Measures

#### 2.4.1. Demographics and Clinical Characteristics

Children’s demographics (i.e., sex and age) and clinical characteristics were collected, including diagnosis, types of cancer therapies received, and time since treatment completion.

#### 2.4.2. Levels of PA

PA levels of the survivors were measured using a validated and self-reported scale—the Chinese University of Hong Kong: Physical Activity Rating for Children and Youth (CUHK-PARCY). This single-item PA rating scale is modified from the Jackson Activity Coding [30] and the Godin–Shephard Activity Questionnaire Modified for Adolescents [31]. The survivors were asked to rate their PA levels during an average week on an 11-point scale, ranging from 0 (no exercise at all) to 10 (vigorous exercise almost every day), accounting for frequency, duration, and intensity. Scores of 0–2, 3–6, and 7–10 represent low, moderate, and high PA levels, respectively. Scores ≥ 8 are equivalent to the PA guideline of engaging in moderate-to-vigorous PA daily as recommended by the WHO [32]. Examples of various types of PA were provided, with each activity provided a Metabolic Equivalent of Task (MET) score, based on the updated Ainsworth compendium of physical activities [33]. All activities were categorized as light intensity (<3.0 METs), moderate intensity (3.0–5.9 METs), and vigorous intensity (≥6.0 METs). The scale has been widely used in pediatric oncology populations [34]. It demonstrates a good internal consistency with a content validity index of 90% and satisfactory test–retest reliability of *r* = 0.86 in Chinese survivors of pediatric cancer [34].

#### 2.4.3. Handgrip Strength

HGS was measured to the nearest 0.1 kg using a digital portable hand dynamometer. During the measurement, the survivors were instructed to stand upright with their feet hip-width apart and to keep their arms at right angles and their elbows by their sides. They were asked to apply maximal grip strength, and to repeat the test three times for their left and right hands individually, with at least 30 s of rest between each push. Grip strength was defined as the maximal measured value among the six measurements for both hands.

#### 2.4.4. Peak Expiratory Flow Rates

A mini-Wright Standard Handheld peak flow meter was used to measure the survivors’ peak expiratory flow rate (PEFR). This is a widely available and useful physiological test to assess an individual’s maximal expiratory flow rate during a forceful expiration following full inspiration. The mini-Wright Standard Handheld peak flow meter has been shown to be an accurate and precise device to determine how well an individual’s airway works by measuring one’s PEFR [35,36]. The survivors were instructed to take a deep breath and blow into the mouthpiece as fast, powerfully, and fully as possible in a single exhalation. They were reminded to ensure that a tight seal was maintained between the mouthpiece and lips during each blow. Each participant was asked to repeat the test for three times, and the highest measured value among the three exhalations was recorded.

#### 2.4.5. Quality of Life

QoL was measured by the Chinese version of the Paediatric QoL Inventory 4.0 Generic Core Scale (PedsQL 4.0 generic version). The scale, which has been widely used in pediatric oncology populations [34,37,38], consists of 23 items assessing four domains in relation to physical functioning, emotional functioning, social functioning, and school functioning. Each item is scored on a 5-point Likert scale ranging from 0 (never a problem) to 4 (almost always a problem). The score of each item requires reverse scoring and linear transformation to a 100-point scale (0–100, 1–75, 2–50, 3–25, and 4–0). The total scores range from 0–100, with higher scores representing better QoL. The scale has a high internal consistency (Cronbach’s α = 0.860), and good test–retest reliability (*r* = 0.65–0.81) [39].

### 2.5. Statistical Analysis

Descriptive statistics were used to summarize the participants’ demographic and clinical characteristics. We used the mean and standard deviation (SD) or frequency and percentage to present continuous and categorical data, respectively. We compared demographic and clinical characteristics, overall QoL score, QoL scores of each domain (i.e., physical, emotional, social, and school functioning), HGS, and PEFR between the survivors with low PA levels and those with moderate PA levels using the 2-tailed *t* test for continuous variables and the χ2 test for categorical variables. One-way analysis of variance was performed to compare the PA levels (i.e., CUHK-PARCY scores) based on the survivors’ demographic and clinical characteristics (i.e., sex, diagnosis, therapy received, and time since treatment completion).

Normality of variables with continuous data was assessed using skewness statistics and normality probability plots. Univariate regression analyses were used to explore the associations of demographic and clinical characteristics (i.e., age, sex, diagnosis, therapy received, and time since treatment completion); PA levels; HGS; and PEFR with each domain of QoL (i.e., physical, emotional, social, and school functioning). Associations of PA levels and HGS with each domain of QoL were examined separately, using a series of hierarchical multiple linear regression analyses to adjust for different variables. In these models, three batches of variables were entered successively into the regression models, including (1) demographic and clinical characteristics, i.e., age, sex, diagnosis, therapies received, and time since treatment completion; (2) HGS and PEFR; and (3) PA levels. Dummy variables were created for the independent variables with a nominal or ordinal level of measurement. All statistical analysis was performed using SPSS version 26.0 (IBM Crop., Armonk, NY, USA). All statistical analyses were two-tailed, and statistical significance level was set at *p* < 0.05. Minimal data were missing, as all of the data were from participants who agreed to participate in the study and were willing to complete the set of questionnaires.

## 3. Results

### 3.1. Demographic and Clinical Characteristics of Pediatric Cancer Survivors

A convenience sample of 191 pediatric cancer survivors was recruited for this study. Of these participants, 77 (40.3%) engaged in low PA levels (CUHK-PARCY score = 0–2), and 114 (59.7%) engaged in moderate PA levels (CUHK-PARCY score = 3–6). Table 1 presents the demographic and clinical characteristics of the whole sample and of survivors with low levels of PA and moderate levels of PA. The mean age of the participants was 12.4 (standard deviation [SD] = 2.4). Most of the participants were male (58.1%), with more male than female participants engaged in moderate levels of PA (male vs. female: 64.9% vs. 35.1%; *p* = 0.021). The most common type of cancer was leukemia (37.2%), followed by lymphoma (26.7%) and brain tumor (22.5%). Participants who diagnosed with leukemia engaged in significantly higher levels of PA (low vs. moderate: 18.2% vs. 50.0%; *p* < 0.001), than participants who diagnosed with brain tumors and bone tumors engaged in lower level of PA (brain tumor: low vs. moderate: 35.1% vs. 14.0%; bone tumor: 16.9% vs. 6.1%; *p* < 0.001). The highest proportion of participants received chemotherapy (41.4%) and mixed treatment modalities (24.1%). Among the low-level PA group, the highest proportion (40.3%) received mixed treatment modalities, whereas, among the moderate-level group, the highest proportion (45.6%) received chemotherapy.

Table 2 shows the physical activity levels based on the CUHK-PARCY scores of the participants. According to the CUHK-PARCY scale, the highest proportion of participants (29.8%) engaged in light PA that lasted longer than 20 min once or twice per week, followed by no PA except during physical education lessons (20.4%). There was no participant engaged in a moderate level of PA (CUHK-PARCY score = 7–10). The mean CUHK-PARCY score was 2.8 (SD = 1.4).

Table 3 presents the differences in overall QoL scores, scores of each QoL domain, HGS and PEFR between participants who engaged in low-level PA and moderate-level PA. Compared with participants who engaged in low-level PA, participants who engaged in moderate-level PA reported statistically significant higher overall QoL scores (mean difference = 17.4; 95% CI: 14.4–20.4; *p* < 0.001), higher scores in physical functioning (mean difference = 24.7; 95% CI: 20.9–28.5; *p* < 0.001), emotional functioning (mean difference = 13.3; 95% CI: 9.5–17.1; *p* < 0.001), social functioning (mean difference = 13.2; 95% CI: 9.1–17.3; *p* < 0.001), and school functioning (mean difference = 13.9; 95% CI: 10.3–17.6; *p* < 0.001), as well as greater HGS (mean difference = 6.6; 95% CI: 5.2–8.0; *p* < 0.001) and PEFR (mean difference = 26.9; 95% CI: 15.9–37.9; *p* < 0.001).

### 3.2. Univariate and Hierarchical Multiple Linear Regression Analyses to Examine the Association between PA Level and HGS for each QoL Domain Scores

Results from the univariate linear regression analyses for variables contributing to each QoL domain are shown in Table 4. The results showed that PA levels had significant positive associations with all QoL domains (physical functioning: *β* = 0.678, *p* < 0.001; emotional functioning: *β* = 0.564, *p* < 0.001; social functioning: *β* = 0.488, *p* < 0.001; school functioning: *β* = 0.562, *p* < 0.001). The results also showed that HGS was significantly associated with all QoL domains (physical functioning: *β* = 0.550, *p* < 0.001; emotional functioning: *β* = 0.473, *p* < 0.001; social functioning: *β* = 0.347, *p* < 0.001; school functioning: *β* = 0.477, *p* < 0.001). PEFR was found to be positively associated with the physical functioning (*β* = 0.279, *p* < 0.001) and school functioning (*β* = 0.263, *p* < 0.001) domains.

A series of hierarchical multiple linear regression analyses were performed to determine the extent to which each outcome variable (HGS, PEFR, and PA level) was associated with various QoL domains (Table 5). The results showed that after being fully adjusted for age, sex, diagnosis, therapy received, time since treatment completion, and PEFR, pediatric cancer survivors engaging in higher PA levels had significantly higher scores in all QoL domains, including physical (*β* = 0.543, *p* < 0.001), emotional (*β* = 0.449, *p* < 0.001), social (*β* = 0.434, *p* < 0.001), and school functioning (*β* = 0.407, *p* < 0.001). Greater HGS was associated with better physical (*β* = 0.230, *p* ≤ 0.001), and emotional (*β* = 0.261, *p* ≤ 0.001) functioning. In addition, the results showed that the demographic and clinical characteristics had only a weak influence on the models, explaining levels of variance ranging from 2.6% in the emotional functioning domain to 10.2% in the physical functioning domain. The addition of HGS significantly increased the variance in each QoL domain, with changes in R^2^ of 0.232, 0.198, 0.113, and 0.183 in the physical, emotional, social, and school functioning domains, respectively. The addition of a higher PA level further increased the variance explained in each QoL domain, with changes in R^2^ of 0.173, 0.118, 0.110, and 0.097 in the physical, emotional, social, and school functioning domains, respectively.

## 4. Discussion

Pediatric cancer significantly impacts QoL among children and adolescents with marked reductions in all domains—physical, emotional, social, and school functioning. Enhancing long-term QoL among pediatric cancer survivors is an indispensable component of pediatric survivorship care. To date, the evidence is lacking for an association between PA and HGS with different QoL domains in Chinese pediatric cancer survivors is lacking. In this study, the results showed that higher PA levels were significantly associated with better physical, emotional, social, and school functioning, as well as overall QoL, in this group of survivors. This association remained significant in all QoL domains, even after adjusting for potential confounders, implying a distinctive and attenuated pattern of associations with QoL. Overall, these results are consistent with findings from earlier studies that have investigated the association between PA levels and QoL in cancer patients/survivors [40,41] and in the general population [42,43]. It is plausible that the underlying mechanism for this significant enhancement across all QoL domains is due to the improved physical fitness resulting from the regular adoption and maintenance of PA. Research has suggested that regular PA plays a vital role in the healthy development of the pediatric oncology population by preventing and reducing the risk of morbidity and mortality associated with cancer and its invasive treatments [44]. The wide array of health benefits for this group of survivors extends to improvements in cardiopulmonary fitness, bone density, functional mobility and flexibility, psychosocial well-being, and cognitive functioning [45,46,47,48]. When taken together, the physical, emotional, social, and school functioning domains of QoL have been substantially enhanced with increased levels of PA. Findings from this study also indicated that pediatric cancer survivors with higher PA levels exhibited more favorable physical fitness as reflected by better HGS and PEFR compared with those survivors with lower PA levels.

Our study compared the demographic and clinical characteristics of survivors who engaged in different PA levels. It is worth noting that male survivors were found to have higher PA levels than female survivors. A similar phenomenon has been reported in the general population, indicating that women are less active than men, with global average rates of physical inactivity of 31.7% among women and 23.4% among men [49]. This gender gap in PA levels happens early: a previous study reported that girls experience less enjoyment when engaging in PA and less confidence in their sporting abilities than boys due to sociocultural stereotypes, which then shape their attitudes towards PA into adulthood [50]. Addressing this gender gap by promoting regular PA in female survivors is crucial. For example, healthcare professionals might collaborate with non-governmental organizations to launch multimedia campaigns focusing on inclusive and positive messaging about PA to encourage all survivors to be physically active. Moreover, our study also found that more survivors diagnosed with leukemia were physically active than those diagnosed with solid tumors (i.e., lymphoma, brain tumor, bone tumor, and gonadal germ-cell tumor). Evidence shows that pediatric survivors of solid tumors reported poorer general health status and functional capacity than survivors of leukemia [38], hindering their active participation in PA. Notably, pediatric brain tumor survivors in this study had significantly lower levels of PA than survivors of leukemia, lymphoma, and gonadal germ-cell tumors. This may be largely attributable to the neurological deficits induced by the invasive treatment, resulting in poor body coordination, loss of balance, and decreased muscle strength [51,52], all of which may impede pediatric brain tumor survivors’ participation in regular PA.

In addition, we found an association between greater HGS and better physical and emotional functioning domains of QoL in pediatric cancer survivors. This might be explained by an improved functional capacity and independence in activities of daily living due to the increase in HGS, which, in turn, enhanced physical and emotional functioning in pediatric cancer survivors. These results are also supported by prior studies conducted in other populations, including adult cancer survivors [53], children and adolescents with idiopathic arthritis [27], and the elderly [25], showing that participants with increased HGS had better QoL scores, particularly in the physical and emotional functioning domains. Recent studies have revealed that greater HGS was associated with lower odds of psychological problems, such as depression and anxiety among elderly adults [54,55]. Based on these findings, future intervention studies might focus on incorporating different types of PA that could maximize survivors’ handgrip strength.

We found that a high proportion of pediatric cancer survivors were engaging in suboptimal amounts of PA. Based on the CUHK-PARCY scoring guideline, 40.3% (77/191) of the participants engaged in low PA levels (i.e., no activity at all or no PA except physical education classes). Only 59.7% (114/191) of participants engaged in moderate PA levels (i.e., apart from the physical education classes, the survivors participated in light PA [MET = 3] with a frequency ranging from once/twice every week to almost every day; or they participated in moderate PA [MET = 5] that lasted longer than 20 min once/twice every week). The results suggested that the levels of PA engagement of the Chinese pediatric cancer survivors were far below the WHO recommended 60 min of moderate to vigorous PA per day [32]. The findings from the present study were in line with a previous study assessing the PA levels of 128 Hong-Kong-based Chinese pediatric cancer survivors, showing that most Chinese survivors (92.2% vs. 100% in the present study) did not adhere to the WHO recommended PA guideline [32]. Declining PA among Chinese pediatric cancer survivors requires prompt attention from healthcare professionals. Comparable findings were obtained in a cohort study including 7287 Western pediatric cancer survivors who survived for 5 years or more [56]. The study found that approximately 47.5% of survivors did not meet the WHO guideline for PA, and the decline in PA was associated with the presence of chronic musculoskeletal problems [56]. Considering the health benefits associated with PA, it is alarming that Chinese pediatric cancer survivors were found to engage in critically lower levels of PA than their Western counterparts.

Physical inactivity in pediatric cancer survivors is an emerging concern in light of the ample evidence suggesting that such inactivity has a detrimental impact on survivors’ health and survival in terms of reducing the functional capacity of most organ systems, aggravating cancer- and treatment-related late effects, and increasing the risk of all-cause mortality [44]. Cardiopulmonary disease [57], lipid abnormalities [58], muscle atrophy and catabolism, and osteoporosis [59] were among the late effects associated with physical inactivity within this population. Conversely, PA has demonstrated rehabilitative and therapeutic value as a treatment for cancer and, indeed, for many chronic medical diseases and conditions. However, the major focus of pediatric cancer survivorship care in Hong Kong’s healthcare system is to provide physiological treatments along the continuum of care by providing long-term regular medical follow-ups to monitor survivors’ physiological condition and to detect any secondary relapses or malignancies after the completion of treatment [37]. There is a paucity of survivorship care that promotes regular PA for this particularly vulnerable group in Hong Kong.

Our findings may have wide-ranging implications for survivorship care delivery and clinical research. Survivors of pediatric cancer may be markedly impaired in multiple QoL domains due to the cumulative burden of disease associated with cancer and its therapies. Mitigation of cancer- and treatment-related late effects and enhancement of long-term health and QoL in pediatric cancer survivors are a priority in the care of this vulnerable group. Success in this regard requires a paradigm shift in the current cancer-care continuum in Hong Kong in terms of providing holistic survivorship care that promotes and sustains positive health behaviors (i.e., regular PA) among pediatric cancer survivors. The findings from the present study further support the beneficial and protective role of regular PA across different QoL domains in pediatric cancer survivors. Furthermore, provided the compelling evidence that health-promoting behaviors, including regular PA, initiated in early childhood persist into adulthood [60], it is imperative for the healthcare professionals to maximize PA among pediatric cancer survivors from the early phase of their survivorship and consequently to improve their QoL. The findings from the present study may better inform clinical guidelines and healthcare paradigms, providing insights into future research and survivorship care planning for this vulnerable group. In particular, interventions designed to promote regular adoption and maintenance of PA should be tailored to female and solid tumor survivors. Importantly, follow-up of the sample in the present study may provide important evidence regarding the long-term beneficial outcomes of engaging in regular PA among Chinese pediatric cancer survivors.

This study has some limitations. First, it was a cross-sectional study; thus, we cannot make any inferences about causality. Longitudinal cohort studies with multiple follow-up time points are warranted to further investigate the potential long-term beneficial effects of regular PA on the QoL and the physical fitness (i.e., HGS and PEFR) of pediatric cancer survivors along their survivorship trajectory. Second, the use of convenience sampling might have limited the generalizability of the results to all Asian pediatric cancer survivors, despite the sample having been obtained from multiple centers. Data from the Hong Kong Cancer Registry showed that, on average, approximately 200 cases of pediatric cancer are diagnosed annually, suggesting that the findings of our study of 191 Hong Kong Chinese pediatric cancer survivors may be representative of the Hong Kong population. Another limitation was that although this study adopted a validated and reliable self-reported PA rating scale to evaluate the participants’ PA levels, potential reporting bias may exist. Further studies might consider using objective measures, such as pedometers and accelerometers, to assess PA levels.

## 5. Conclusions

To conclude, in pediatric cancer survivors, adoption of healthy lifestyle behaviors, i.e., participating in regular PA, is positively associated with better QoL in the physical, emotional, social, and school domains, as well as in overall QoL. There is thus a critical need to explore effective approaches to promoting regular PA in pediatric cancer survivors.

## Figures and Tables

**Table 1:** Demographic and clinical characteristics of pediatric cancer survivors by level of physical activity (*n* = 191).

Characteristics	Levels of PA			*p*		
	All	Low-Level Group	Moderate-Level Group		CUHK-PARCY Scores	*p*
	(*n* = 191)	(*n* = 77, 40.3%)	(*n* = 114, 59.7%)		Mean ± SD	
Age, mean ± SD, year	12.4 ± 2.4	12.6 ± 2.5	12.3 ± 2.4	0.388		
Sex, n (%)				0.021		0.014
Male	111 (58.1)	37 (48.1)	74 (64.9)		3.04 ± 1.28	
Female	80 (41.9)	40 (51.9)	40 (35.1)		2.54 ± 1.48	
Diagnosis				<0.001		<0.001
Leukemia	71 (37.2)	14 (18.2)	57 (50.0)		3.45 ± 1.24	
Lymphoma	51 (26.7)	22 (28.6)	29 (25.4)		2.75 ± 1.32	
Brain tumor	43 (22.5)	27 (35.1)	16 (14.0)		2.23 ± 1.17	
Bone tumor	20 (10.5)	13 (16.9)	7 (6.1)		1.95 ± 1.47	
Gonadal germ-cell tumor	6 (3.1)	1 (1.3)	5 (4.4)		3.33 ± 1.37	
Therapy received				0.001		0.004
Surgery	30 (15.7)	10 (13.0)	20 (17.5)		3.17 ± 1.58	
Chemotherapy	79 (41.4)	27 (35.1)	52 (45.6)		2.84 ± 1.28	
Radiotherapy	15 (7.9)	4 (5.2)	11 (9.6)		3.00 ± 1.51	
Bone marrow transplantation	21 (11.0)	5 (6.5)	16 (14.0)		3.48 ± 1.54	
Mixed treatment modalities	46 (24.1)	31 (40.3)	15 (13.2)		2.24 ± 1.12	
Time since treatment completion				0.985		0.675
6–12 months	74 (38.7)	31 (40.3)	43 (37.7)		2.88 ± 1.44	
13–24 months	27 (14.1)	10 (13.0)	17 (14.9)		2.74 ± 1.16	
25–36 months	21 (11.0)	8 (10.4)	13 (11.4)		3.19 ± 1.54	
37–48 months	17 (8.9)	8 (10.4)	9 (7.9)		2.47 ± 1.13	
49–60 months	37 (19.4)	14 (18.2)	23 (20.2)		2.84 ± 1.50	
>60 months	15 (7.9)	6 (7.8)	9 (7.9)		2.60 ± 1.24	

Abbreviations: CUHK-PARCY, The Chinese University of Hong Kong: Physical Activity Rating for Children and Youth; PA, physical activity; SD, standard deviation.

**Table 2:** Pediatric cancer survivors’ PA levels based on CUHK-PARCY scores (*n* = 191).

CUHK-PARCY Scores	Description	All	Male	Female
		(*n* = 191)	(*n* = 111, 58.1%)	(*n* = 80, 41.9%)
0	No physical activity at all, spend most of your time sitting or sleeping.	4 (2.1)	1 (0.9)	3 (3.8)
1	No physical activity except little physical activity during physical education lessons.	34 (17.8)	13 (11.7)	21 (26.3)
2	No physical activity except being active during physical education lessons.	39 (20.4)	24 (21.6)	15 (18.8)
3	Besides the physical education classes, I participate in light activities that last longer than 20 min only once or twice every week.	57 (29.8)	32 (28.8)	25 (31.3)
4	Besides the physical education classes, I participate in light activities that last longer than 20 min for 3 times a week	35 (18.3)	28 (25.2)	7 (8.8)
5	Besides the physical education classes, I participate in light activities that last longer than 20 min almost every day.	15 (7.9)	10 (9.0)	5 (6.3)
6	Besides the physical education classes, I participate in moderate activities that last longer than 20 min only once or twice every week.	7 (3.7)	3 (2.7)	4 (5.0)
7	Besides the physical education classes, I participate in moderate activities that last longer than 20 min 3 to 5 times every week.	0 (0)	0 (0)	0 (0)
8	Besides the physical education classes, I participate in moderate activities that last longer than 20 min almost every day.	0 (0)	0 (0)	0 (0)
9	Besides the physical education classes, I participate in vigorous activities that last longer than 20 min 3 times or less every week.	0 (0)	0 (0)	0 (0)
10	Besides the physical education classes, I participate in vigorous activities that last longer than 20 min almost every day.	0 (0)	0 (0)	0 (0)
		Mean ± SD		
		2.8 ± 1.4	3.04 ± 1.28	2.54 ± 1.48

Abbreviation: CUHK-PARCY, The Chinese University of Hong Kong: Physical Activity Rating for Children and Youth; 0–2: low physical activity; 3–6: moderate level of physical activity; 7–10: high level of physical activity; SD, standard deviation.

**Table 3:** Comparison of the QoL scores, HGS and PEFR across each domain between participants engaged in low PA level and moderate PA level.

Variables, mean ± SD	Levels of PA				
	All	Low-Level Group	Moderate-Level Group	Mean Difference, 95% CI	*p*
	(*N* = 191)	(*n* = 77, 40.3%)	(*n* = 114, 59.7%)		
Overall PedsQL score	77.2 ± 13.3	67.3 ± 10.9	83.8 ± 10.3	17.4, 14.4–20.4	<0.001
Physical functioning domain score	76.1 ± 17.7	62.2 ± 14.9	85.5 ± 12.6	24.7, 20.9–28.5	<0.001
Emotional functioning domain score	77.7 ± 14.4	69.9 ± 12.8	83.0 ± 12.9	13.3, 9.5–17.1	<0.001
Social functioning domain score	79.3 ± 15.4	71.9 ± 14.6	84.3 ± 13.9	13.2, 9.1–17.3	<0.001
School functioning domain score	76.2 ± 14.3	68.3 ± 12.4	81.4 ± 13.0	13.9, 10.3–17.6	<0.001
HGS, kg	18.4 ± 5.7	14.4 ± 3.6	21.0 ± 5.4	6.6, 5.2–8.0	<0.001
PEFR, L/min	126.5 ± 39.6	110.2 ± 33.8	137.6 ± 39.6	26.9, 15.9–37.9	<0.001

Abbreviation: PA, physical activity; SD, standard deviation; CI, confidence interval; PedsQL, Paediatric QoL Inventory 4.0 Generic Core Scale; HGS, handgrip strength; PEFR, peak expiratory flow rate.

**Table 4:** Univariate linear regression to investigate the variables contributing to each PedsQL domain of pediatric cancer survivors.

	Physical Functioning Domain			Emotional Functioning Domain			Social Functioning Domain			School Functioning Domain		
	*β*	*SE B*	*p*	*β*	*SE B*	*p*	*β*	*SE B*	*p*	*β*	*SE B*	*p*
Age	−0.028	0.527	0.699	−0.066	0.426	0.364	−0.118	0.456	0.105	−0.764	0.424	0.446
Sex	−0.113	2.592	0.121	−0.436	2.111	0.663	−0.003	2.270	0.966	−0.151	2.076	0.038
Diagnosis	−0.263	0.978	<0.001	−0.206	0.803	0.004	−0.228	0.859	0.004	−0.225	0.795	0.002
Therapy received	−0.160	0.877	0.027	−0.101	0.716	0.165	−0.037	0.773	0.612	−0.109	0.711	0.133
Time since treatment completion	0.147	0.709	0.042	0.045	0.580	0.536	0.073	0.622	0.318	−0.066	0.576	0.367
PA levels	0.678	0.686	<0.001	0.564	0.624	<0.001	0.488	0.709	<0.001	0.562	0.621	<0.001
HGS	0.550	0.188	<0.001	0.473	0.160	<0.001	0.347	0.183	<0.001	0.477	0.159	<0.001
PEFR	0.279	0.031	<0.001	0.190	0.026	0.008	0.135	0.028	0.062	0.263	0.025	<0.001

Abbreviations: HGS, handgrip strength; PEFR, peak expiratory flow rate; PA, physical activity; PedsQL, Paediatric QoL Inventory 4.0 Generic Core Scale; *SE B*, standard error of unstandardized coefficient; *β*, standardized coefficient.

**Table 5:** Hierarchical linear regression analyses to investigate the associations of the level of PA and HGS with QoL scores in each domain (*n* = 191).

		Physical Functioning Domain			Emotional Functioning Domain			Social Functioning Domain			School Functioning Domain		
		*β*	*SE B*	*p*	*β*	*SE B*	*p*	*β*	*SE B*	*p*	*β*	*SE B*	*p*
Model 1	Age	−0.011	0.515	0.874	−0.044	0.434	0.555	−0.098	0.462	0.184	−0.003	0.427	0.963
	Sex	−0.128	2.528	0.071	−0.026	2.130	0.724	0.033	2.265	0.646	−0.135	2.092	0.064
	Diagnosis	−0.221	0.997	0.002	−0.182	0.840	0.015	−0.228	0.893	0.002	−0.194	0.825	0.009
	Therapy received	−0.157	0.871	0.029	−0.078	0.734	0.292	0.074	0.781	0.316	−0.085	0.721	0.250
	Time since treatment completion	0.186	0.702	0.010	0.067	0.591	0.366	0.079	0.629	0.283	−0.037	0.581	0.615
		R^2^ = 0.125 Adjusted R^2^ = 0.102			R^2^ = 0.052 Adjusted R^2^ = 0.026			R^2^ = 0.072 Adjusted R^2^ = 0.047			R^2^ = 0.076 Adjusted R^2^ = 0.051		
	
Model 2	Age	−0.070	0.448	0.257	−0.100	0.392	0.137	−0.140	0.439	0.045	−0.054	0.387	0.412
	Sex	−0.024	2.230	0.696	0.066	1.951	0.325	0.102	2.185	0.147	−0.041	1.927	0.541
	Diagnosis	−0.120	0.876	0.058	−0.090	0.767	0.187	−0.159	0.859	0.026	−0.105	0.757	0.122
	Therapy received	−0.100	0.757	0.107	−0.023	0.662	0.727	0.116	0.741	0.097	−0.036	0.654	0.585
	Time since treatment completion	0.160	0.607	0.010	0.047	0.477	0.477	0.065	0.595	0.348	−0.062	0.525	0.348
	PEFR	0.044	0.030	0.505	−0.013	0.853	0.853	−0.027	0.029	0.718	0.075	0.026	0.292
	HGS	0.490	0.211	<0.001	0.475	<0.001	<0.001	0.366	0.207	<0.001	0.416	0.182	<0.001
		R^2^ = 0.358 Adjusted R^2^ = 0.333 R^2^ change = 0.232			R^2^ = 0.250 Adjusted R^2^ = 0.221 R^2^ change = 0.198			R^2^ = 0.185 Adjusted R^2^ = 0.154 R^2^ change = 0.113			R^2^ = 0.259 Adjusted R^2^ = 0.231 R^2^ change = 0.183		
	
Model 3	Age	−0.008	0.388	0.874	−0.049	0.364	0.435	−0.091	0.413	0.167	−0.008	0.365	0.896
	Sex	0.000	1.915	0.998	0.086	1.799	0.165	0.121	2.040	0.065	−0.023	1.805	0.716
	Diagnosis	−0.014	0.773	0.794	−0.003	0.726	0.967	−0.075	0.823	0.272	−0.026	0.728	0.693
	Therapy received	−0.063	0.651	0.238	0.007	0.612	0.906	0.146	0.694	0.027	−0.008	0.614	0.892
	Time since treatment completion	0.176	0.521	0.010	0.061	0.489	0.325	0.078	0.555	0.230	−0.050	0.491	0.417
	PEFR	−0.018	0.026	0.758	−0.064	0.024	0.334	−0.076	0.027	0.277	0.029	0.024	0.668
	HGS	0.230	0.206	<0.001	0.261	0.193	<0.001	0.158	0.219	0.053	0.222	0.194	0.005
	PA level	0.543	0.852	<0.001	0.449	0.800	<0.001	0.434	0.908	<0.001	0.407	0.803	<0.001
		R^2^ = 0.530 Adjusted R^2^ = 0.510 R^2^ change = 0.173			R^2^ = 0.368 Adjusted R^2^ = 0.340 R^2^ change = 0.118			R^2^ = 0.295 Adjusted R^2^ = 0.264 R^2^ change = 0.110			R^2^ = 0.356 Adjusted R^2^ = 0.328 R^2^ change = 0.097		
	

Abbreviations: HGS, handgrip strength; PEFR, peak expiratory flow rate; PA, physical activity; PedsQL, Paediatric QoL Inventory 4.0 Generic Core Scale; *SE B*, standard error of unstandardized coefficient; *β*, standardized coefficient.

## Data Availability

The data presented in this study are available on request from the corresponding author. The data are not publicly available due to privacy and ethical concerns.
